# Metabolic Plasticity and Inter-Compartmental Interactions in Rice Metabolism: An Analysis from Reaction Deletion Study

**DOI:** 10.1371/journal.pone.0133899

**Published:** 2015-07-29

**Authors:** Rahul Shaw, Sudip Kundu

**Affiliations:** 1 Department of Biophysics, Molecular Biology and Bioinformatics, University of Calcutta, 92 APC Road, Kolkata 700009, West Bengal, India; 2 Centre of Excellence in Systems Biology & Biomedical Engineering, (TEQIP, Phase-II), University of Calcutta, 92 APC Road, Kolkata 700009, West Bengal, India; University of Georgia, UNITED STATES

## Abstract

More than 20% of the total caloric intake of human population comes from rice. The expression of rice genes and hence, the concentration of enzymatic proteins might vary due to several biotic and abiotic stresses. It in turn, can influence the overall metabolism and survivability of rice plant. Thus, understanding the rice cellular metabolism, its plasticity and potential readjustments under different perturbations can help rice biotechnologists to design efficient rice cultivars. Here, using the flux balance analysis (FBA) method, with the help of *in-silico* reaction deletion strategy, we study the metabolic plasticity of genome-scale metabolic model of rice leaf. A set of 131 reactions, essential for the production of primary biomass precursors is identified; deletion of any of them can inhibit the overall biomass production. Usability Index (I_*U*_) for the rest of the reactions are estimated and based on this parameter, they are classified into three categories—maximally-favourable, quasi-favourable and unfavourable for the primary biomass production. The lower value of 1 − I_*U*_ of a reaction suggests that the cell cannot easily bypass it for biomass production. While some of the alternative paths are energetically equally efficient, others demand for higher photon. The variations in (i) ATP/NADPH ratio, (ii) exchange of metabolites through chloroplastic transporters and (iii) total biomass production are also presented here. Mutual metabolic dependencies of different cellular compartments are also demonstrated.

## Introduction

Rice is a socio-economically important food grain for more than half of the world’s human population [1]. However, this rice plant, like other cereal crops is also under several biotic and abiotic stresses [2]. In addition, while the human population is increasing, the available cultivable lands and water are declining. Thus, the challenge is to design an efficient stress-tolerant more nutritious high-yielding rice cultivar. Researchers are trying to meet the challenges in different ways like breeding approach, identification of stress-related genes, changing the anatomy of rice leaf, etc. Since metabolism plays a central role in cellular process and the metabolic engineering is proven as a very useful technique for over/less production of desired metabolites or improvement of cellular properties in several species [3, 4], attempts have been made to model and analyze the genome-scale metabolism of rice [5].

The metabolic modeling technique can be grouped into two categories—(i) kinetic modeling and (ii) structural modeling. Since, the kinetic modeling requires details of enzyme kinetic data, the technique is usually limited to systems having small number of reactions. On the other hand, the correct stoichiometry and the thermodynamic feasibility of the biochemical reactions are required for structural modeling. Thus, the structural modeling technique is used in genome-scale metabolic modeling and its analysis. A number of genome-scale metabolic models (GSMs) of various organisms, including bacteria [6], simplest eukaryote [7] and plants [5, 8, 9] are available. The flux balance analysis based on the linear programming (LP) method is normally used to analyze these genome-scale metabolic models.

Any species including rice plant is exposed to several environmental conditions, which might change the expressions of cellular proteins including enzymatic genes. The down-regulation of an enzymatic gene’s expression can result several consequences, such as (i) no change in the efficiency of the corresponding reaction, (ii) a partial blockage in enzymatic reaction’s activity, or (iii) a full blockage (i.e., the reaction becomes inactive). The partial or full blockage of an enzymatic gene’s activity can change the metabolism of a cell. It can alter the metabolic phenotype, such as the composition and amount of the biomass precursors it synthesizes in normal condition. Whether this alteration of metabolic phenotype would be lethal or not depends on the necessity of the biomass precursors. Alternatively, the cell does not exhibit any change in metabolic phenotype and finds some alternative metabolic pathways to produce the same amount of biomass it produces in normal condition. It has been already shown that a living species has enough metabolic flexibility to adapt itself to different biotic and abiotic perturbations [10, 11]. This metabolic flexibility of a cell to readjust its metabolism for survival in various conditions is referred as metabolic plasticity.

Deletion of a reaction from a metabolic network effectively generates a different metabolic genotype. There are two possible biological means by which a reaction can be deleted from a metabolic network; those include (i) the enzyme(s) associated with that particular reaction is knocked out (assuming they have only catalytic activities) and (ii) severe down-regulation of the associated gene(s) so that the reaction becomes inactive. Here, we have studied the effect of reaction deletion (the full blockage of the enzymatic reaction) in a genome-scale metabolic model of rice leaf [5]. In specific, we have not considered any change in metabolic phenotype (biomass is kept fixed in experimentally observed proportion), rather, this work aims to understand the inherent flexibility of the metabolic network to produce the biomass when a change in the metabolic genotype occurs. The reactions, deletion of which cause inhibition to the synthesis of biomass, are considered as ‘essential’. Among rest of the reactions, some can be easily bypassed by the cellular metabolic system to synthesize the biomass; while the others cannot. To find whether a reaction is frequently or occasionally favoured by the cellular metabolic process to produce the cellular biomass under a given constraint, Barve et al. (2012) [12] have used an expression, termed as ‘superessentiality index’ of a reaction. They generated 500 random networks (each of the networks is able to produce biomass of *E. coli*) from an universal network of 5906 reactions and determined the essentiality of each of the reactions. The reactions required in all metabolic networks were defined as ‘superessential’ (extremely essential). The superessentiality index of a reaction was defined as a ‘fraction of networks in which the reaction is essential’. The value of superessentiality index helps one to understand whether a reaction can be easily bypassed by any known metabolic pathway or not. We have used this concept to derive a mathematical expression, termed as the Usability Index (see Methods for its description) for the reactions those are not extremely essential (‘non-essential’) for biomass production.

The rice genome-scale metabolic model is used here (i) to find the essential reactions for biomass production, (ii) to classify the rest of the reactions based on their Usability Index and (iii) to observe the metabolic plasticity of rice metabolism. Further, we have analyzed how the deletion of any reaction can affect the overall cellular economy in terms of photon demand. It should be mentioned that the incident photon is the primary source of a photosynthetic organism’s ATP and NADPH productions which, in turn, are used in fixing the inorganic nutrients into biomass through a series of chemical reactions. Consequently, for the deletions of some of the reactions, the variation in ATP/NADPH ratio is also observed. How this variation is readjusted within the cellular metabolic system has also been discussed with a few examples. We also report the effects of reaction deletion on total amount of biomass production and exchange of metabolites through chloroplastic transporters. Inter-compartmental interactions within a cell play an indispensable role for any eukaryotic organism. This reaction deletion study elucidates how the metabolism of different compartments interact with each other. In summary, here, different genotypes in a rice cell is created through *in-silico* reaction (hence, gene) deletion strategy and the cellular responses are simulated to observe the possible active metabolic states.

## Materials and Methods

### Metabolic Model

Here, the partially compartmentalized genome-scale metabolic model of rice (*Oryza sativa*) [5] is used. It consists of 1733 reactions (including 42 biomass precursors) and 1484 metabolites distributed into three compartments—chloroplast, mitochondria and cytosol. It is the first photosynthetic model of any plant leaf cell which can produce primary biomass precursors (amino acids, nucleotides, lipid, starch, sucrose, cellulose and lignin) in experimentally determined proportions, taking the inorganic nutrients and light (photon) energy. Input light energy is represented by an artificial photon transporting (as incident photon flux) reaction (${}^{Chl}P_{tx}$) in the chloroplast of the cell. These photons are utilized in the photophosphorylation reactions (light cyclic and non-cyclic) present in the chloroplast. The stoichiometries of these two reactions are same as described in Poolman et al. (2013) [5]. It should be mentioned that these reactions actually occur in thylakoid membrane and they are deduced from elementary flux mode analysis of a detailed model of photophosphorylation [13]. Here, the demand of photon refers to the minimum amount of photon (value can be obtained from the flux of ${}^{Chl}P_{tx}$ transporter) required to produce the biomass using the cellular biochemical machinery present in the model.

The stoichiometric matrix **S**_*m* × *n*_ of this metabolic network is constructed, where *m* is the number of metabolites and *n* is the number of reactions. At steady state, **S.v** = 0, where **v** is the flux vector of the reactions. Lower bound of the flux for an irreversible reaction is set to 0 and upper bound of the flux can take any positive value decided by the mathematical process. For reversible reactions, the lower and upper bounds of fluxes can take any negative and positive values, respectively. Fluxes of primary biomass precursors (amino acids, nucleotides, starch, sucrose, cellulose and lignin) are fixed in experimentally observed proportions as used in Poolman et al. (2013) [5]. During the simulation, nutrient (ammonia, nitrate, carbon dioxide, phosphate and sulfate) uptake, photon consumption and oxygen released could take any flux value.

FBA is used to find steady state fluxes of the reactions which reflect the flow of metabolites into the network [14]. FBA method utilizes linear programming (LP) to optimize the objective function to get the possible solution of metabolic space. Here, the objective function is the sum of the fluxes of all reactions and minimization of this objective function is used here as the optimization criteria. Minimization of the total cellular flux assumes that the cell tries to optimize its cellular economy and it has been proved to be an effective cellular objective for genome-scale studies on plant metabolism [5, 8].

### Reaction Deletion

A deleted reaction (complete inhibition) in the metabolic model refers to a cellular state where the corresponding enzyme is not able to catalyze the respective reaction. Here, either the associated genes do not express in sufficient amounts to catalyze the reaction or they do not express at all in that condition or have been knocked out. We should mention that there is no linear relationship between enzymatic gene expression and the associated reaction’s flux, so it is not rational to predict the flux based solely on relative abundances of gene expression data [15].

Here, each of the reactions of rice metabolic network is deleted [completely inhibited; termed here as mutant type (MT) network] and the metabolic response is simulated by FBA to test whether the modified metabolic network (i.e., MT network) is able to produce the biomass in experimentally observed fixed proportions or not. If the simulation can not find any possible solution, the reaction is termed as essential for biomass production. An essential reaction must carry a non-zero flux to produce the cellular biomass; however, it does not refer that the rest of the reactions are not at all important to produce the biomass. Rather, some of them might frequently take part in biomass production while others do not. Thus, to determine the importance of a single reaction in overall metabolism of rice leaf cell, Usability Index (I_*U*_) is used. I_*U*_ is calculated using the following equation.

$$\begin{array}{c} I_{U}=\frac{Number\;of\;times\;a\;reaction\;participates\;in\;the\;metabolism\;when\;others\;are\;deleted\;(one\;at\;a\;time)}{Total\;number\;of\;non–essential\;reactions\;-1} \end{array}$$ (1)

The value of I_*U*_ helps to understand the importance of a reaction in different metabolic genotypes under a given constraint. Here, the higher value of I_*U*_ of a reaction suggests that the cell favours this reaction to produce its biomass components. When FBA is allowed to use all the reactions present in the model (no reaction is deleted; enzymes associated with all the reactions are assumed to be present in sufficient amount to catalyze the reactions), the simulated state is referred here as the wild type (WT) state. MT state is obtained by the deletion of an individual reaction. Thus, each MT refers to a specific mutant genotypic state of the cell. The fluxes through a reaction in WT and MT may be different. So the fold change (FC) in flux for a particular reaction from WT to MT is calculated using the equation given below.

$$\begin{array}{c} FC=\frac{f_{MT}-f_{WT}}{f_{WT}}\times 100\% \end{array}$$ (2)

Here, f_*WT*_ and f_*MT*_ are the fluxes of the same reaction in WT and MT solutions, respectively.

### Metabolic Modeling Tool Used

All computational implementations are done using ScrumPy metabolic modeling tool [16] and results are analyzed by scripts written in Python (www.python.org).

## Results and Discussion

The genome-scale metabolic model of rice leaf is used here to simulate the metabolic responses under the genotypic perturbations created by the complete inhibition of individual reaction. Since primary aim of this work is to understand the metabolic flexibility, we have first identified the essential reactions; without any of them, the cell is not able to produce the primary biomass in fixed proportion. Then, I_*U*_ is calculated for each of the reactions to understand how much this reaction is favourable by the metabolic system. Subsequently, the photon usage efficiency of the alternative metabolic pathways are compared with that of the WT. The analysis also includes (i) some examples to show the variation in ATP and NADPH usages, (ii) changes in the transport of C_3_ compound between chloroplast and cytosol through chloroplastic transporters and (iii) changes in total biomass production at a fixed value of incident photon. Then, the flux responses are analyzed to show that change in fluxes of some reactions in one compartment can influence the fluxes of reactions in other compartments.

### Essential Reactions and Metabolic Plasticity in Rice Metabolic Network

A set of 131 reactions is found to be essential for biomass production (S1 Dataset). Among them, 11 reactions are present in chloroplast and 7 are in mitochondria. While studying the effect of knockout of enzymatic genes in C_3_ plant *Arabidopsis thaliana*, Wang et al. (2012) reported that nearly 10% of reactions are essential for biomass production [17]. Here, the results show that nearly 8% of the total reactions are essentials in rice metabolic network. This essential set includes several known important reactions. For example, the results show that the light dependent non-cyclic photophosphorylation is an essential reaction as it produces both ATP and NADPH using light energy (photon). RuBisCo catalyzes the fixation of CO_2_ and its deletion shows complete inhibition of biomass production. The metabolic network in the absence of phosphoribulokinase (chl_Ru5Pk; EC 2.7.1.19) is not able to produce the biomass precursors in experimentally observed proportions. These are consistent with realistic behaviour of plant’s cellular biochemistry [18]. Part of the TCA cycle involved in the synthesis of 2-oxoglutarate remains active in all solutions, and this indicates its importance in biomass production. It is already established that 2-oxoglutarate is an important precursor for several amino acid biosynthetic pathways in plant and regulating the coordination of C and N metabolism [19]. The results show that most of the essential reactions are involved in biosynthesis of biomass precursors, e.g., HISTOLDEHYD-RXN for histidine biosynthesis, ASPARTATEKIN-RXN for lysine biosynthesis, etc. It is expected, and also reported for *E. coli* that most of the biosynthetic pathways’ reactions are essential [12]. The essential reactions in this study are associated with 226 genes locus ids are presented in S1 Dataset. Thus, the deletions of these reactions or the associated genes would inhibit the biomass production and hence, might be lethal to the plant.

### Importance of Reactions Based on Usability Index

The reactions other than the essential are termed as non-essential reactions for primary biomass production. Nonessentiality of a reaction does not mean that the cell does not utilize this reaction to produce the biomass. Rather, it indicates that there exist alternative possible routes within the cellular metabolism using which the cell can produce same amount of the biomass. In addition, these alternative routes might be important for a specific perturbed state caused due to environmental changes to which the cell is exposed. Under some perturbed conditions, the knockout of enzymatic reaction associated gene(s) results in complete blockage of the associated reaction. The different states of genes (knockout or knock-down) represent different cellular genotypes, and respective metabolic responses could relate to the active cellular metabolic states.

**Fig 1:**
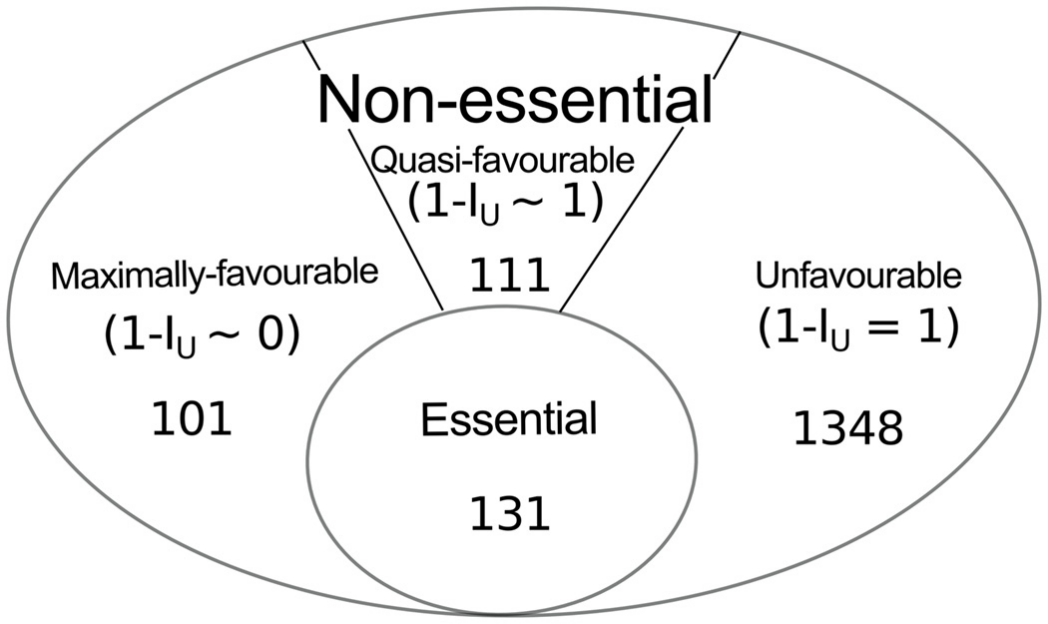
Categorization of reactions depending on their importance in experimentally observed biomass production. A total of 1691 reactions are shown excluding 42 fixed biomass precursors.

The Usability Index helps to understand how much these reactions are favoured by the cell or how easily these reactions can be bypassed. The value of 1 − I_*U*_ indicates how easily a reaction will be bypassed when a perturbed or mutant condition arises. The non-essential reactions are classified here into three categories—‘maximally-favourable’, ‘quasi-favourable’ and ‘unfavourable’. The 1 − I_*U*_ ∼ 0 value of a reaction indicates that the probability of bypassing this reaction is less and these reactions are termed as maximally-favourable for biomass production. The reactions with 1- I_*U*_ ∼ 1 are defined as quasi-favourable, i.e., they are unused most of the time; however, they might be activated for driving metabolism in rare circumstances. Unfavourable reactions are those for which 1 − I_*U*_ = 1. It should be noted that all these categorization of metabolic reactions are done in fixed biomass (experimentally observed proportion) condition. We have identified that 101 (1 − I_*U*_ ∼ 0.0006 to 0.077), 111 (1 − I_*U*_ ∼ 0.923 to 0.999) and 1348 (1 − I_*U*_ = 1) reactions are maximally-favourable, quasi-favourable and unfavourable for biomass production, respectively (Fig 1, S2 Dataset).

A large set of reactions (78%) is found to be unfavourable and this result indicates that most of the routes in the entire metabolic network are not used to produce biomass precursors considered here; but they might be active to produce other biomass components including the synthesis of secondary metabolites. On the other hand, an inference could be made by the presence of a large number of maximally-favourable and quasi-favourable reactions that the plasticity of rice metabolic network is very high.

### Metabolic Plasticity and its Relation to Photon Consumption

Deletion of a large number of reactions does not inhibit the production of biomass and this indicates the metabolic plasticity of the cell as well as the robustness of cellular biochemical mechanisms. Consequently, this suggests that there are alternative routes the plant can use when needed. However, it does not ensure that all the alternative routes are equally economic, i.e., the cell needs equal amount of energy to produce the biomass using these alternative pathways. This energy, indeed, comes from the photon absorbed and is utilized in biochemical machinery.

**Fig 2:**
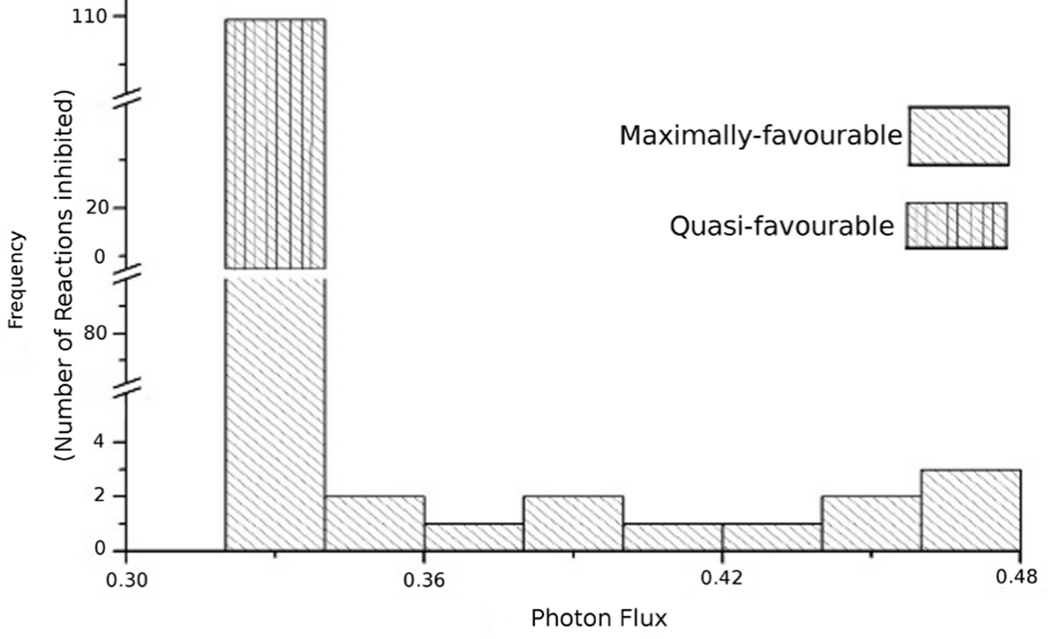
Histogram of minimum photon flux needed while deleting maximally and quasi-favourable reactions. Each reaction is deleted and photon demand for this perturbation is measured through FBA.

**Fig 3:**
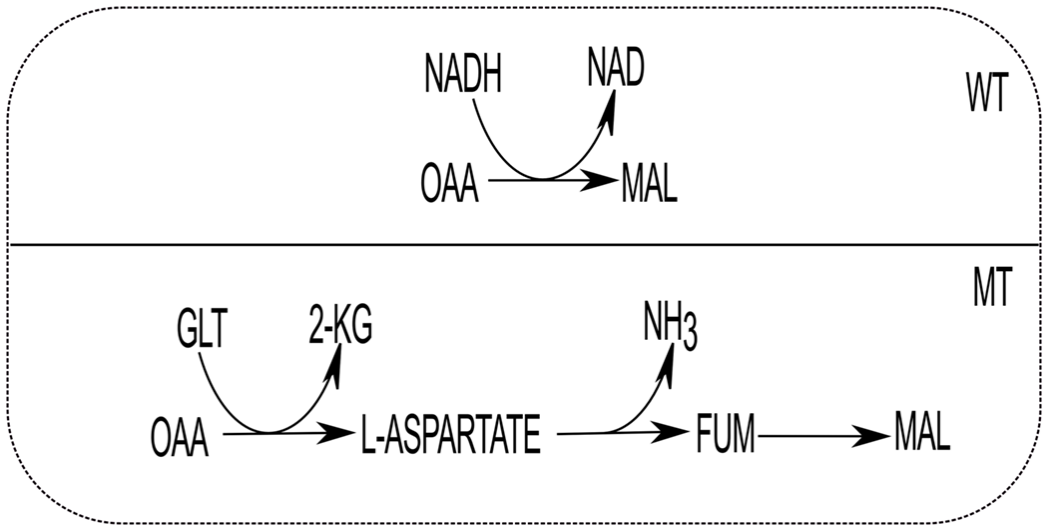
Adaptation of alternate metabolic route due to deletion effect. An example of OAA to MAL synthesis is shown. WT is the path preferred by the cellular metabolism in wild type condition to produce malate in cytosol. Mutant type (MT) shows the alternative route of the WT. Here, GLT indicates glutamate.

While a reaction is blocked and the metabolic network finds at least one alternative route (bypassing the blocked reaction and taking another path to produce corresponding biomass; FBA finds the suitable alternate while searching through all of the possibilities), the photon fold change (FC in ${}^{Chl}P_{tx}$) is calculated for this shift. While deletion of some of the maximally-favourable reactions can find alternative routes utilizing same amount of photon as used by WT, the demands of photons for others vary between 0.32 to 0.48 light flux unit (Fig 2). Table 1 shows some maximally-favourable reactions whose alternative routes are also energy efficient in terms of photon demand. One might argue that why the back-up routes of the maximally-favourable reactions are not used where the energy demand for their elimination is same? A reason of this is that while the photon demands are same for both the WT and MT pathways (FC of ${}^{Chl}P_{tx}$ ∼ 0), the sum of fluxes of the participating reactions in the WT (as reflected in overall objective function) is lowest. This lowest value actually corresponds to the most efficient cellular economy of a cell [5]. For instance, when cytosolic malate dehydrogenase is deleted (shown in Fig 3, WT path), the MT path takes the route OAA (oxaloacetate) → L-ASPARTATE → FUM (fumarate) → MAL (malate). Here, the 1-I_*U*_ index for cytosolic malate dehydrogenase (MALATE-DEH-RXN, a maximally-favourable reaction) is 0.0019. The deletion of this reaction causes no change in photon demand, but the total flux of cellular objective function in MT solution space becomes higher (6.2% fold change in total flux) than the WT. So, using this WT path, cell can maximize its overall cellular economy. Further, there is always a possibility of having different solution for a particular objective. The presence of these cannot affect our main observation; rather, it suggests higher metabolic plasticity of cellular metabolism.

**Table 1:** Some of the reactions with low (1 − I_*U*_) values and negligible photon fold change are shown here. These reactions can be bypassed by the cellular metabolism to produce biomass. ‘chl_’ indicates a chloroplastic reaction; while the rests are cytosolic. From top to bottom the reactions are xylulose 5 phosphate isomerase, ribose-5-phosphate isomerase, glycine-aminotransferase, acetaldehyde dehydrogenase and O-acetylhomoserine sulfhydrylase.

Reaction Deleted	In Pathway	Photon Flux FC	(1 − I_*U*_)
chl_X5Piso	Calvin cycle	0	0.0006
chl_R5Piso	Calvincycle	0	0.0006
GLYCINE-AMINOTRANSFERASE-RXN	Photorespiration	0	0.0006
ACETALD-DEHYDROG-RXN	Mixed acid fermentation	0.0608	0.0006
ACETYLHOMOSER-CYS-RXN	Homocysteine biosynthesis	0.0035	0.0019

As the unfavourable reactions (1 − I_*U*_ = 1) do not take part in biomass production, photon demand remains unchanged due to their deletions. Interestingly, the deletions of quasi-favourable reactions (1 − I_*U*_ ∼ 1) also do not show any change in photon demand. It suggests that quasi-favourable reactions can be easily bypassed and can have several alternatives to produce the biomass.

**Fig 4:**
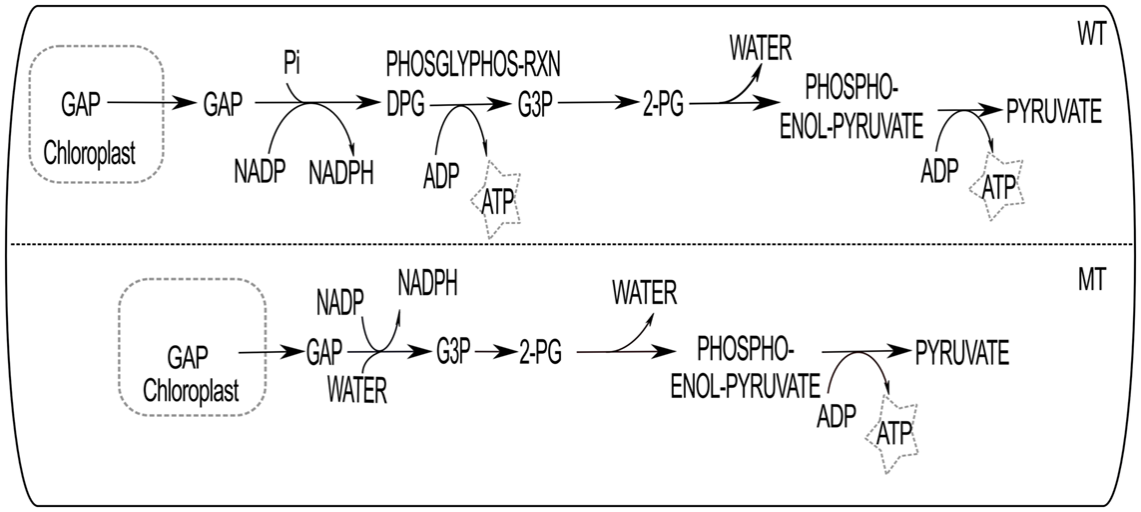
The deletion effect of PHOSGLYPHOS-RXN is shown here. In WT, GAP (glyceraldehyde 3-phosphate) transported from chloroplast to cytosol and converted to DPG (1,3-bisphospho-D-glycerate). DPG to G3P (3-phospho-D-glycerate) and 2-PG (2-phospho-D-glycerate) conversion takes place and ultimately produces pyruvate to be utilized in mitochondria. MT is mutant type path when the reaction PHOSGLYPHOS-RXN is deleted.

**Fig 5:**
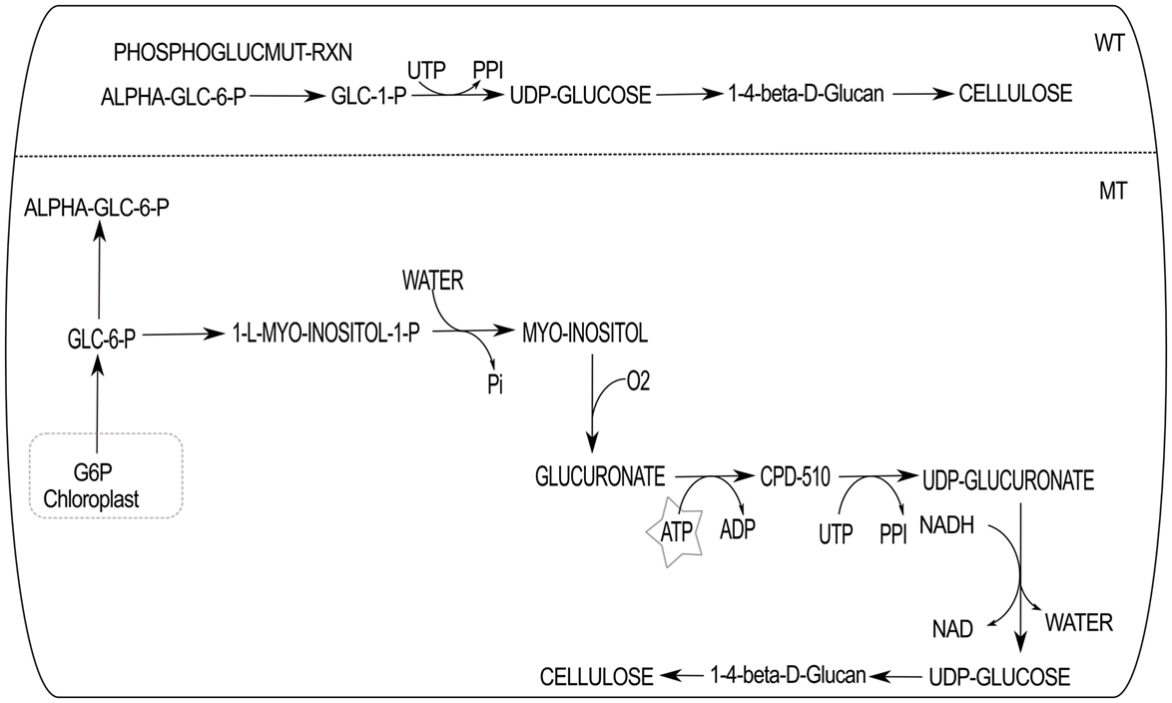
The deletion effect of PHOSPHOGLUCMUT-RXN is shown here. The WT shows the pathway by PHOSPHOGLUCMUT-RXN to synthesize cellulose. While deleted PHOSPHOGLUCMUT-RXN, the alternative possible route via myo-inositol pathway to produce cellulose is shown in MT.

We have observed that there are 13 maximally-favourable reactions (1 − I_*U*_ ∼ 0) which show photon FC > 1 when deleted (S2 Dataset). Nine of them are presented in Table 2 for further discussions. Absence of any of them diverts the cellular metabolism into higher energy demanding pathways. Higher FC in photon usage can occur by two ways: (i) either the removed reaction is used to generate energy [ATP or NADP(H)] or (ii) its deletion causes diversion into energy demanding paths (thus, higher photon demanding paths) as shown in the Figs 4 and 5, respectively.

**Table 2:** Fold Change (FC) in photon flux while some of the maximally-favourable reactions (1 − I_*U*_ ∼ 0) are deleted. ‘chl_’ and ‘mit_’ represent the chloroplastic and mitochondrial reactions, respectively. From top to bottom the reactions are mitochondrial complex V, I, chloroplastic glyceraldehyde 3-phosphate dehydrogenase, phosphoglycerate kinase, mitochondrial malate dehydrogenase, complex III, IV, cytosolic phosphoglycerate kinase and phosphoglucomutase. While in case of LightNonCyc reaction, the increase or decrease in fold change is presented; since light cyclic (LightCyc) is not utilized in WT; only the values of fluxes are presented here.

Reaction Deleted	Photon (${}^{Chl}P_{tx}$) (FC)	LightCyc (Flux)	LightNonCyc (FC)	ATP/NADPH	% biomass production possible with WT photon (MT_*fp*_)
mit_Complex_V	47.47	0.0168	−26.27	3.0	40.5%
mit_Complex_I	47.47	0.0168	−26.27	3.0	40.5%
chl_G3Pdh	39.09	0.00	39.09	1.28	58.3%
chl_PGK	39.09	0.00	39.09	1.28	58.3%
mit_MalDH	35.88	0.006	9.22	1.7	57.7%
mit_Complex_III	24.31	0.00	24.31	1.28	67.9%
mit_Complex_IV	24.31	0.00	24.31	1.28	67.9%
PHOSGLYPHOS-RXN	12.79	0.00	12.79	1.28	83.9%
PHOSPHOGLUCMUT-RXN	6.09	0.00	6.09	1.28	94.3%

### Some of the Alternative Routes and Readjustment of ATP and NADPH Usage

**Fig 6:**
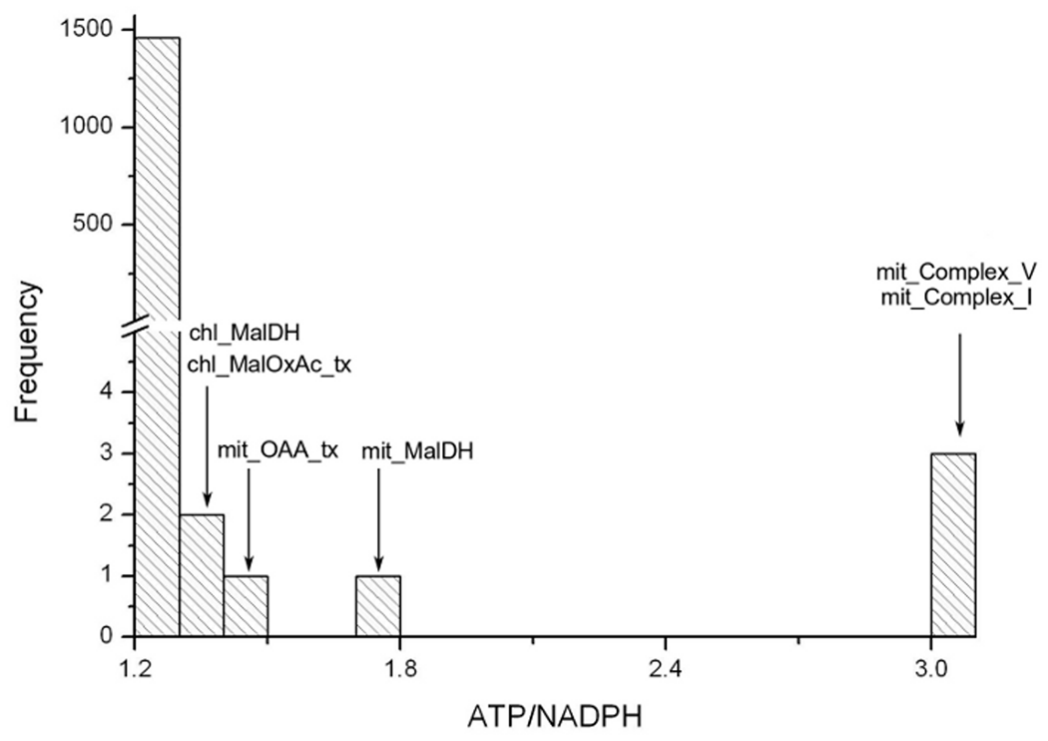
Variation of ATP/NADPH ratio due to deletions of reactions. ATP and NADPH produced in the light reactions are calculated by the their stoichiometric coefficient times the flux of the light reactions. Deletions of a few mitochondrial and chloroplastic reactions (shown by down arrow) increase the ATP/NADPH ratio.

Several MT metabolic states need higher amount of energy as well as reductant to maintain the cellular processes required for biomass production in the absence of favourable reactions (Figs 2 and 6). This section demonstrates how the cell readjusts its metabolism using alternative biochemical pathways (and consequently generates and utilizes different amounts of ATP and NADPH) optimizing the cellular economy.

While the DPG (1,3-bisphospho-D-glycerate) to G3P (3-phospho-D-glycerate) producing reaction (PHOSGLYPHOS-RXN) by enzyme phosphoglycerate kinase [20, 21] is inhibited (Fig 4), 12.79% increment of photon usage is observed. This enzyme, produces ATP from ADP; thus deletion of this reaction forces the system to use alternative reactions to produce the biomass and also to meet the ATP requirement. The results suggest that in economically favourable condition and in sufficient light intensities, the MT cell favours to adjust photon usage to meet the ATP demand. As an alternative mechanism, cell may favour to choose reaction 1.2.1.9-RXN that produces GAP (glyceraldehyde-3-phosphate) to G3P by cytosolic glyceraldehyde 3-phosphate dehydrogenase (GAPC; activity of GAPC is reported in plant [22]). GAPC is an important enzyme which plays a significant role in water deficit condition and stomatal sensitivity to abscisic acid in *Arabidopsis thaliana* [23]. It is evident from Table 2 that the deletion of PHOSGLYPHOS-RXN increases the flux of light non-cyclic reaction which produces ATP as well as NADPH. However, the reaction PHOSGLYPHOS-RXN generates only ATP. So, the extra amount of NADPH produced by relatively higher flux, through light non-cyclic reaction, must be utilized somewhere in MT path. In fact, the reaction 1.2.1.13-RXN is active in the WT and produces NADPH and DPG from GAP, NADP and phosphate. This reaction becomes inactive in the MT.

Another example is illustrated in Fig 5. Here, the enzyme phosphoglucomutase converts ALPHA-GLC-6-P (*α*-glucose 6-phosphate) to GLC-1-P (glucose 1-phosphate) in the WT and this pathway synthesizes cellulose. The UDP-glucose pyrophosphorylase, an important enzyme for cellulose deposition, catalyzes glucose-1-phosphate and UTP to produce UDP-glucose and pyrophosphate (PPI) [24]. When the phosphoglucomutase associated reaction (PHOSPHOGLUCMUT-RXN) is deleted, the cellulose synthesis pathway diverts from the WT; and one of the possible pathway it can follow in MT is presented in Fig 5. However, the MT path is high energy demanding which causes 6% increase in photon usage. The participating reaction here produces 1-L-MYO-INOSITOL-1-P (myo-inositol 1-phosphate) from GLC-6-P (glucose 6-phosphate) by the enzyme myo-inositol-3-monophosphate synthase. Inositol oxygenase playing an important role for cell wall biosynthesis in plant [25], catalyzes myo-inositol to glucuronate in MT cellulose biosynthesis path. Glucuronokinase (EC 2.7.1.43) catalyzes an energy consuming reaction to produce CPD-510 (glucuronate 1-phosphate) from glucuronate. This reaction needs ATP which causes MT path much more energy demanding than the WT. Glucuronate-1-phosphate uridylyltransferase (EC 2.7.7.44) produces UDP-glucuronate and PPI from glucuronate 1-phosphate and UTP (uridine-triphosphate). The UDP-glucuronate is further converted to UDP-glucose which leads to cellulose biosynthesis. These WT and MT paths show internal plasticity in plant metabolism to survive in the perturbed conditions. It is also known that the myo-inositol 1-phosphate synthase (EC 5.5.1.4) plays an important role for inositol biosynthesis and it is found to be essential for growth in plants [26].

It is observed that while any one of the complexes I, III, IV and V in the mitochondrial electron transport chain is deleted, the metabolic process needs significantly higher amount of photon flux to supply ATP for biomass production. It is evident from Table 2 that the deletion of mitochondrial complexes I and V are higher photon demanding than complexes III and IV. In addition, the deletion of mitochondrial malate dehydrogenase shows a higher photon demand (FC = 35.88%); on the other hand, the deletion of chloroplastic malate dehydrogenase does not show any significant change in photon demand.

In chloroplast, glyceraldehyde 3-phosphate dehydrogenase catalyzes a step in Calvin cycle by conversion of glyceraldehyde-3-phosphate (GAP) to glycerate 1,3-bisphosphate (BPGA). This reaction is also associated with NADPH production. Thus, removal of this reaction causes an increase in photon flux to produce the biomass in fixed proportion. Similarly, the deletion of another adjacent reaction catalyzed by phosphoglycerate kinase (EC 2.7.2.3) converting PGA (3-phosphoglycerate) to BPGA in the same path causes same photon FC. The light dependent non-cyclic (LightNonCyc in our reaction list) reaction remains active all the time; however, in a few cases, slight decrement in its flux is observed which is compensated mostly by light dependent cyclic reaction. The decrease in NADPH produced by light non-cyclic reaction is tuned by other NADPH producing sources. Alternative routes for deletions of some of the maximally-favourable reactions are also presented in S1 Table.

Rice leaf metabolism under varying light shows that photorespiration can salvage the excess energy produced at higher light intensities [5]. Consequently, it is suggested that the inhibition of photorespiration might not always be beneficial [5]. The results also indicate that the plant might use different routes or pathways to readjust the metabolism at varying incident photon [5]. However, a detailed analysis is not performed to understand the effect of reaction deletion. Here, we have observed that due to blockage of some of the metabolic reactions, the cell finds alternative pathways to produce the biomass using higher amount of photon flux. This confirms our previous observation [5] that the plant has the potentiality to use alternative paths to salvage the excess energy generated at higher light intensities. Moreover, examples shown here demonstrate that the use of alternative pathway helps the cellular metabolism in balancing the cellular redox and ATP utilization.

### Variation of ATP/NADPH Ratios in Light Reactions

Photosynthetic organisms need NADPH as the reducing power for several carbon and nitrogen fixing metabolic processes occurring within the cell. ATP is the essential currency metabolite, needed in many metabolic processes as a supply of energy. The utilization of ATP and NADPH in a cell depends on the condition in which the cell is being exposed [27]. The light cyclic and non-cyclic photophosphorylation reactions are the primary source of generation of ATP and NADPH. It is evident from Fig 6 that deletions of a large number of reactions do not alter ATP/NADPH ratio. On the other hand, this ratio varies in a few cases. For example, this ratio is different for the deletions of chloroplastic and mitochondrial malate dehydrogenase. The deletions of mitochondrial complexes I and V cause production of more ATP through light reactions, thus alter the ATP/NADPH ratio generated by the light cyclic and non-cyclic reactions. The variation of ATP/NADPH ratios for deletions of some of the maximally-favourable reactions are also presented in Table 2.

### Exchange of Metabolites Through Intracellular Transporters

The intracellular transporters of a plant cell transport metabolites from one compartment to another. There are a number of chloroplastic transporters which exchange Pi (inorganic phosphate) and specific metabolites (carbon compounds) between chloroplast and cytosol. For example, the C_3_ compound (PGA, GAP and DHAP (dihydroxyacetone phosphate)), generated in the Calvin cycle within the chloroplast, can be transported to the cytosol with an exchange of cytosolic Pi through three chloroplastic transporters, namely, PGA, GAP and DHAP transporters. Thermodynamically, these exchange reactions are reversible, i.e., they can also transport cytosolic PGA, GAP and DHAP to the chloroplast with an exchange of chloroplastic Pi.

**Fig 7:**
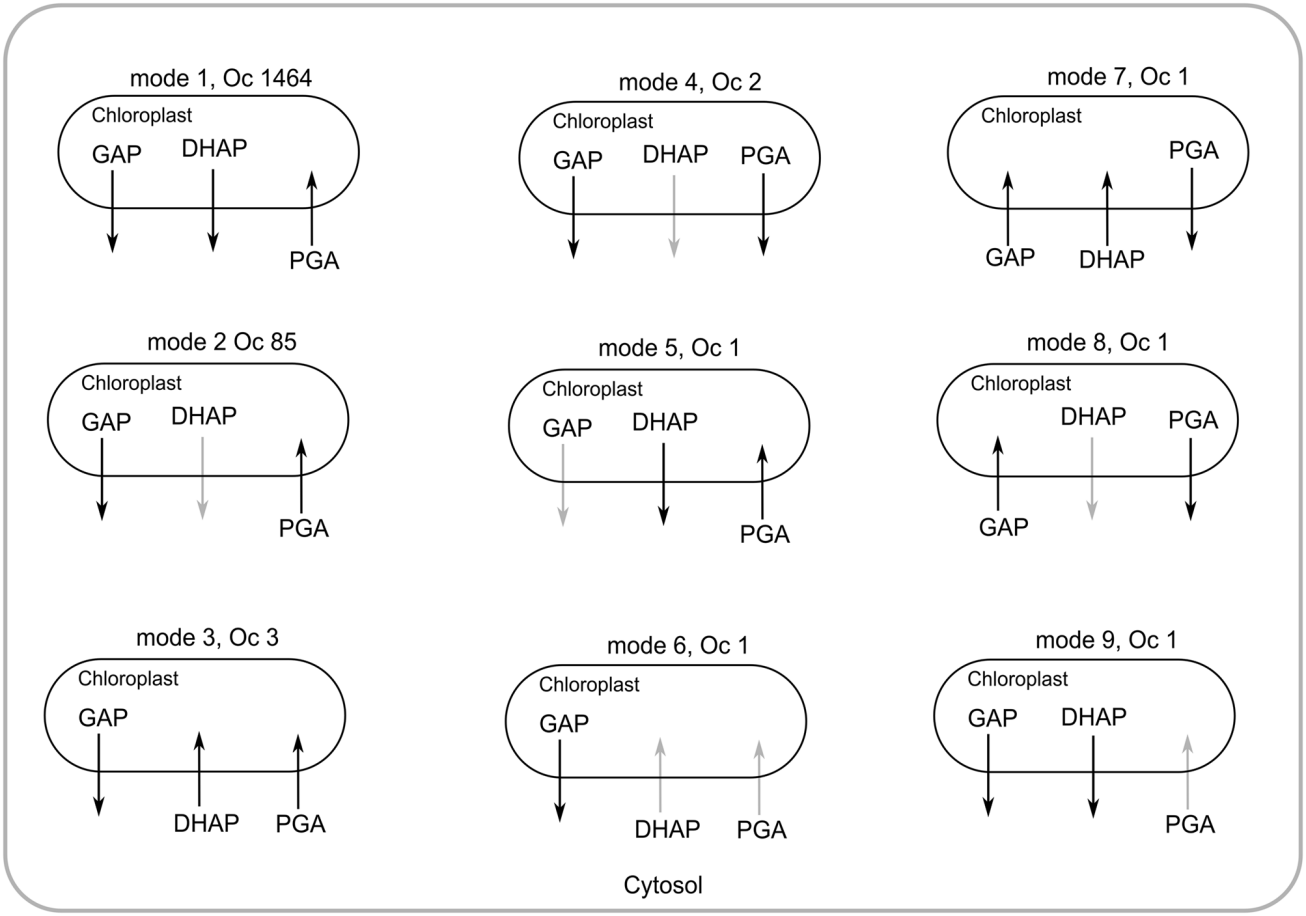
Different modes of the chloroplastic C_3_ compound transporters (GAP, DHAP and PGA) observed due to deletions of reactions. ‘Oc’ referred to the number of occurrence of the particular mode in the total simulation. Faded arrow represents the inactive transporter. Mode 1 (WT mode) occurs most of the time, where chloroplastic GAP and DHAP are transported to the cytosol and cytosolic PGA is transported to the chloroplast.

We have deleted all the non-essential reactions one by one and analyzed the exchange of metabolites through these chloroplastic transporters. The results are presented in Table 3. Moreover, the possible different active modes of chloroplastic C_3_ transporters are shown in Fig 7. In 1464 cases, the chloroplastic GAP and DHAP are transported to cytosol and cytosolic PGA is transported to chloroplast with Pi exchange. However, chloroplastic DHAP transporter becomes inactive in 89 cases. In four cases, chloroplastic PGA is transported to the cytosol and the cytosolic GAP is transported twice to the chloroplast. On the other hand, in most of the cases, chloroplastic GAP and cytosolic PGA are transported to the cytosol and chloroplast, respectively.

**Table 3:** Flux reversibility count for chloroplastic transports. It represents the thermodynamic possibility of inorganic phosphate exchange.

Transporters	Number of times the transporter carries positive flux (Exporting Pi to cytosol)	Number of times the transporter carries negative flux (Importing Pi into chloroplast)
chl_PGA_tx	1553	4
chl_GAP_tx	2	1556
chl_DHAP_tx	4	1466
chl_G6P_tx	6	1464
chl_PEP_tx	1	3
chl_Ru5P_tx	7	1546
chl_E4P_tx	1	1552

In the WT, the chloroplastic PGA transporter transports PGA from cytosol to the chloroplast. When any one of the mitochondrial MalDH (malate dehydrogenase), cytosolic phosphoglycerate kinase, chloroplastic G3Pdh (glyceraldehyde 3-phosphate dehydrogenase) or PGK (phosphoglycerate kinase) reactions is blocked, the chloroplastic PGA transporter transports chloroplastic PGA to the cytosol. In WT, the G6P (glucose 6-phosphate) is produced in the chloroplast and transported to the cytosol. The transport becomes reverse when any one of the chloroplastic FBPase (fructose 1,6-bisphosphatase), G3Pdh, Ald1 (Aldolase A), R5Piso, PGI (phosphoglucose isomerase) or PGK reactions is blocked. While chloroplastic G3Pdh or PGK reaction is blocked, the GAP transporter transports GAP from cytosol to the chloroplast. The DHAP transporter exports Pi from chloroplast to cytosol when any one of the chloroplastic triosephosphate isomerase, ribose-5-phosphate isomerase, PGK or cytosolic fructose-bisphosphate aldolase catalyzed reactions is blocked.

### Reaction Deletion, Photon Demand and Biomass Production

The minimum amount of photon needed in WT to produce the biomass in experimentally fixed proportion is ∼ 0.32 light flux unit. However, deletions of some reactions require higher amount of incident photon for the same purpose. Here, the incident photon flux is kept fixed at ∼ 0.32 light flux unit (as obtained in WT); then a reaction is deleted and the cellular metabolism is simulated to predict the fluxes through the biomass precursors. This mutant type state, simulated in fixed photon flux is denoted as MT_*fp*_. This gives an estimate of how much biomass can be generated in mutant type using the amount of photon needed in WT. It is expected that MT_*fp*_ would generate a lower amount of biomass using WT photon. It is clear from Table 2 that deletions of some reactions reduce the biomass production when the photon is kept fixed at ∼ 0.32 light flux unit. For example, deletions of chloroplastic PGK and mitochondrial malate dehydrogenase reactions cause significant decrease in biomass production. MT_*fp*_ solutions for these constraints produce 58.3% and 57.7% of total biomass, respectively. It indicates that inhibition of some maximally-favourable reactions can end up with reduced biomass in lower light.

### Inter-Compartmental Link

**Fig 8:**
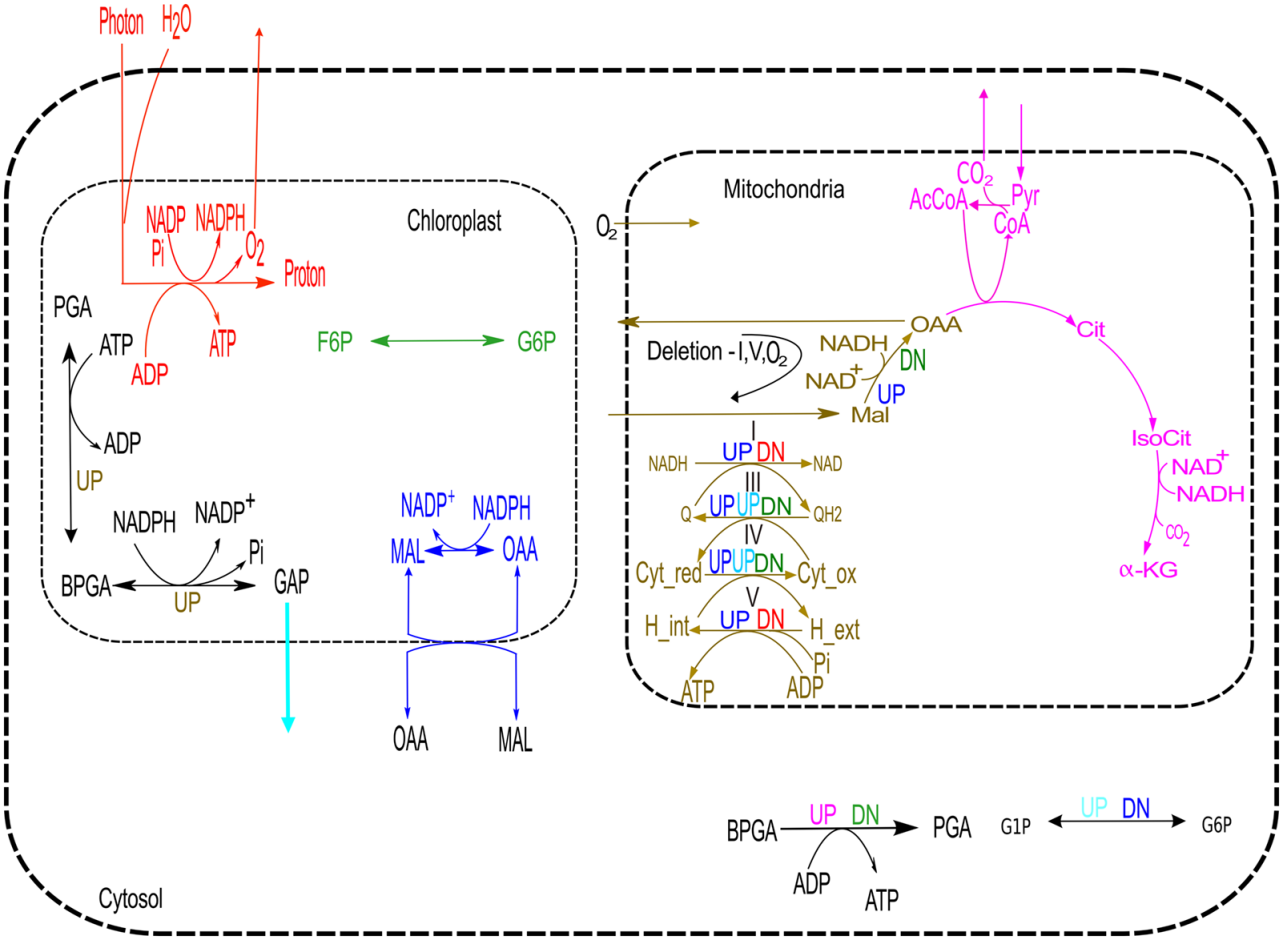
Up/down regulation of reactions/paths of one compartment when a reaction from another compartment is deleted. Reactions marked with UP/DN are deleted; and the color of UP/DN shows the flux increase (UP) or decrease (DN) in another compartment. For instance, when mit_MalDH is deleted, the chloroplastic malate oxaloacetate shuttle (represented by blue UP) up regulates and the F6P to G6P conversion (represented by green DN) in chloroplast down-regulates. Malate oxaloacetate shuttle in mitochondria becomes reverse of the shown direction when complexes I, V or mitochondrial O_2_ is removed. Although deletion of the reactions shown here causes high up/down regulation in flux in the same compartment or in cytosol but here we focused to observe maximum flux FC in different compartment i.e., change in mitochondria when chloroplastic reaction is deleted and vice versa. Here, CoA, Pyr, IsoCit, *α*-KG, AcCoA, Cit, Cyt_red, Cyt_ox, Q, QH2, MalOxAc, _ext and _int indicate Coenzyme A, Pyruvate, isocitrate, alpha ketoglutarate, Acetyl-CoA, citrate, cytochrome c reductase, cytochrome c oxidase, ubiquinone, ubiquinol, malate oxaloacetate, external and internal, respectively.

It is already known that the chloroplastic and mitochondrial metabolisms are highly dependent on each other and they serve some important cellular functions including ATP production and regulation of redox state in the cell [28–30]. Here, we have observed that the deletions of any of the mitochondrial electron transport chain reactions (mitochondrial complexes I, III, IV and V) show larger flux changes in the reaction associated with photon and consequently, these are associated with changes in the light non-cyclic and some times cyclic reactions (Table 2). Further, deletion of some other mitochondrial and cytosolic reactions also influence the fluxes of some of the chloroplastic reactions. The observations are presented in Fig 8 and summarized below.

#### Deletion Effect of Mitochondrial Reactions

Deletions of complexes I and V in mitochondria up-regulate malate-oxaloacetate shuttle in chloroplast and down-regulate light dependent non-cyclic reaction. In addition, the light dependent cyclic reaction becomes active. The deletions of complexes III and IV up-regulate malate-oxaloacetate shuttle, light non-cyclic reaction and GAP transport from chloroplast and down-regulate F6P (fructose 6-phosphate) to G6P conversion. Deletion of malate-dehydrogenase (Mal → OAA) in mitochondria up-regulates malate-oxaloacetate conversion, light non-cyclic reaction in chloroplast and down-regulates F6P to G6P production.

#### Deletion Effect of Chloroplastic Reactions

While the reactions catalyzed by chloroplastic PGK (PGA → BPGA) and G3Pdh (BPGA → GAP) are blocked, the fluxes in electron transport chain (ETC) are increased along with malate to oxaloacetate shuttle in mitochondria.

#### Deletion Effect of Cytosolic Reactions

Elimination of cytosolic BPGA to PGA conversion reaction up-regulates some reactions of TCA cycle (shown in pink in Fig 8) and down-regulates the F6P to G6P conversion in chloroplast. Further, when the reaction converting G1P (glucose 1-phosphate) to G6P in cytosol is deleted, the flux of GAP from chloroplast to cytosol is increased and the flux through malate-oxaloacetate shuttle in chloroplast is decreased. In addition, the flux through the light non-cyclic reaction is also increased in both the cases.

Thus, our results clearly demonstrate that the plant metabolism has interactions between different cellular compartments. It is already reported that the plant mitochondria has enough flexibilities to generate different amount of ATP utilizing the truncated TCA cycle and ETC [31]. It is also suggested that depending on plant’s cellular need, different biochemical modes can be active. Further, there exist beneficiary interactions between photosynthetic carbon assimilation in chloroplast and mitochondrial metabolism [32, 33]. Thus, the changing metabolic dynamics among the different compartments help the plant to meet it’s cellular and metabolic demand.

## Summary and Future Scope

Starting with a partially compartmentalized rice leaf genome-scale metabolic model, here, the flexibility of its metabolism is studied. In specific, a set of essential reactions (without any of these, plant is unable to produce the biomass in experimentally determined fixed proportion) is identified. The rest of the reactions are classified based on the condition that whether these reactions can be easily bypassed or they are favourable by the cellular metabolism. The photon demands of the metabolic system while using alternative paths are calculated. Some of these alternative pathways are economic in terms of photon demand, while others are not. The readjustments of metabolism to utilize the cellular ATP and NADPH through some alternative pathways are also discussed. While the biomass production remains constant, the higher photon demanding pathways might represent active metabolic states of the plant at higher light intensities. The effect of reaction deletion on (i) exchange of metabolites through chloroplastic transporters and (ii) the interactions of the metabolism of different compartments are also demonstrated.

However, the present version of the rice metabolic model is partially compartmentalized. Thus, one can include other compartments and the relevant reactions in the model and extend the analysis to understand the underlying mechanism of cellular metabolism. Likewise, the system can be studied under other cellular objectives (e.g., biomass or ATP maximization, etc.). Rice plant also produces several secondary metabolites and inclusion of those into the model might increase the number of essential reactions. However, despite the possibility of these improvements, this study should provide a platform for the rice biotechnologists to design stress-tolerant and efficient rice cultivars.

## Supporting Information

S1 Dataset List of essential reactions with pathway and gene (locus id) information. (XLS)

S2 Dataset List of maximally-favourable, quasi-favourable and unfavourable reactions with corresponding Usability Index and photon fold change (FC). (XLS)

S1 Table Alternative metabolic pathways, variation in ATP/NADPH ratio and biomass production for deletions of some of the maximally-favourable reactions. (PDF)
