# Supplementary material for: Metabolic Plasticity and Inter-Compartmental Interactions in Rice Metabolism: An Analysis from Reaction Deletion Study
Source: PLoS One. 2015 Jul 29;10(7):e0133899. doi: 10.1371/journal.pone.0133899 (PMC4519304; doi:10.1371/journal.pone.0133899)
Supplement: S1 Table — (PDF) [file pone.0133899.s003.pdf]

1 Supporting Information: Alternative paths of some of the maximally-favourable reactions

Table I: While any of the maximally-favourable reactions is deleted, the cell finds alternative known plant specific metabolic pathways by which it can produce the biomass. However, when the input photon is kept fixed at the value (∼0.32 light flux unit) required in WT plant cell, the total amount of biomass produced in the MT_{fp} decreases.

Reaction Deleted	Comment	Alternative reactions	Comment	ATP/NADPH in light reactions	% biomass production possible with WT photon (MT_{fp})
UDPKIN-RXN	In Tobacco, after heat shock, reaction catalysed by nucleoside diphosphate kinase is inhibited [1].	THIOREDOXIN-REDUCT-NADPH-RXN	Involved in oxidative stress response [2]	1.28	97.1%
		RXN-6182	Present in plant [3]		
		TRIOSEPISOMERIZATION-RXN	Present in plant [4]		
		UDPREDUCT-RXN	Induction of AtRNR2A is likely required in Arabidopsis for the replicative stress checkpoint (www.brenda-enzymes.org)		
		DUDPKIN-RXN	NDPK2 and H2O2 are involved in salt resistance (www.brenda-enzymes.org)		

Table II: Continuation of the table I.

Reaction Deleted	Comment	Alternative reactions	Comment	ATP/NADPH in light reactions	% biomass production possible with WT photon (MT_{fp})
chl_PGK	Limitation of Pi supply to the isolated spinach chloroplasts limits ATP synthesis and as a consequence reduction of 3-phosphoglycerate to triose phosphate is inhibited via mass action effects on 3-phosphoglycerate kinase [5].	RXN-8089	Present in plant (www.brenda-enzymes.org)	1.28	58.3%
		RXN-6182	Present in plant [6]		
		chl_StPase			
		TRIOSEPISOMERIZATION-RXN	Present in rice [7]		
		PGLUCISOM-RXN	Anaerobic (flooding) responsive enzyme [8]		
		2TRANSKETO-RXN	The activity of transketolase can be moderately increase under salt and oxidative stress [9]		
		5.4.4.2-RXN	Arabidopsis (ICS) (www.brenda-enzymes.org)		
		PHEAMINOTRANS-RXN	Present in plant		
		RXN-1981	Salicylic acid can be synthesized from chorismate by means of isochorismate synthase and it is important for plant defense such as against pathogens [10]		
		RXN-2002	Petunia gene can encode cinnamoyl-CoA hydratase-dehydrogenase (benzoic acid biosynthesis) [11]		
		RXN-7183	Present in rice (www.gramene.org)		
		PREPHENATEDEHYDRAT-RXN	Present in rice		
		CINNAMOYL-COA-REDUCTASE-RXN	Present in rice [12]		
		RIBULP3EPIM-RXN	Present in rice [13, 14]		

Table III: Continuation of the table II.

Reaction Deleted	Comment	Alternative reactions	Comment	ATP/NADPH in light reactions	% biomass production possible with WT photon (MT_{fp})
mit_MalDH	Photosynthetic activity can increase if mitochondrial malate dehydroganase is decreased [15]	mit_SucThioK		1.7	57.7%
		mit_Complex_II			
		GLUTAMATE-DEHYDROGENASE-(NAD(P)+)-RXN-(NAD)	OsGDH2 present in rice (www.brenda-enzymes.org)		
		mit_AlphaKGDH			
		chl_LightCyc			
		PHEAMINOTRANS-RXN	Present in plant [16]		
		RXN-6182			
		TRYPSYN-RXN			
		TRIOSEPISOMERIZATION-RXN			
		ORNITHINE - CYCLODEAMINASE-RXN	Present in plant [17]		
		PREPHENATEDEHYDRAT-RXN	Present in rice (www.brenda-enzymes.org)		
		FUMHYDR-RXN	Present in Arabidopsis [18]		
HOMOSERKIN-RXN	Inhibition is studied in radish leaf [19]	RXN-721	Cystathionine γ-synthase can regulates the carbon flow towards methionine in plants [20]	1.28	98.5%
		PYRUVATE-DECARBOXYLASE-RXN	Pyruvate decarboxylase1 present in Arabidopsis and is required during anoxia [21]		
		PYRUFLAVREDUCT-RXN			
THRESYN-RXN	Down-regulation is studied in transgenic potato plants [22].	CYSPH-RXN	Present in Arabidopsis (www.brenda-enzymes.org)	1.28	98.5%
		TRIOSEPISOMERIZATION-RXN			
		RXN-6182			
		PYRUVATE-DECARBOXYLASE-RXN			
		PYRUFLAVREDUCT-RXN	www.gramene.org		

References

[1] Valenti D, Vacca RA, de Pinto MC, De Gara L, Marra E, Passarella S. In the early phase of programmed cell death in Tobacco Bright Yellow 2 cells the mitochondrial adenine nucleotide translocator, adenylate kinase and nucleoside diphosphate kinase are impaired in a reactive oxygen species-dependent manner. Biochimica et Biophysica Acta (BBA)-Bioenergetics. 2007;1767(1):66–78.

[2] Dos Santos CV, Rey P. Plant thioredoxins are key actors in the oxidative stress response. Trends in Plant Science. 2006;11(7):329–334.

[3] Nowitzki U, Flechner A, Kellermann J, Hasegawa M, Schnarrenberger C, Martin W. Eubacterial origin of nuclear genes for chloroplast and cytosolic glucose-6-phosphate isomerase from spinach: sampling eubacterial gene diversity in eukaryotic chromosomes through symbiosis. Gene. 1998;214(1):205–213.

[4] Kurzok HG, Feierabend J. Comparison of a cytosolic and a chloroplast triosephosphate isomerase isoenzyme from rye leaves: II. Molecular properties and phylogenetic relationships. Biochimica et Biophysica Acta (BBA)-Protein Structure and Molecular Enzymology. 1984;788(2):222–233.

[5] Giersch C, Robinson SP. Regulation of photosynthetic carbon metabolism during phosphate limitation of photosynthesis in isolated spinach chloroplasts. Photosynthesis Research. 1987;14(3):211–227.

[6] Wang R, Okamoto M, Xing X, Crawford NM. Microarray analysis of the nitrate response in Arabidopsis roots and shoots reveals over 1,000 rapidly responding genes and new linkages to glucose, trehalose-6-phosphate, iron, and sulfate metabolism. Plant Physiology. 2003;132(2):556–567.

[7] Xu Y, Hall TC. Cytosolic triosephosphate isomerase is a single gene in rice. Plant Physiology. 1993;101(2):683–687.

[8] Sachs MM, Subbaiah CC, Saab IN. Anaerobic gene expression and flooding tolerance in maize. Journal of Experimental Botany. 1996;47(1):1–15.

[9] Rapala-Kozik M, Kowalska E, Ostrowska K. Modulation of thiamine metabolism in Zea mays seedlings under conditions of abiotic stress. Journal of Experimental Botany. 2008;59(15):4133–4143.

[10] Wildermuth MC, Dewdney J, Wu G, Ausubel FM. Isochorismate synthase is required to synthesize salicylic acid for plant defence. Nature. 2001;414(6863):562–565.

[11] Qualley AV, Widhalm JR, Adebesin F, Kish CM, Dudareva N. Completion of the core β-oxidative pathway of benzoic acid biosynthesis in plants. Proceedings of the National Academy of Sciences. 2012;109(40):16383–16388.

[12] Kawasaki T, Koita H, Nakatsubo T, Hasegawa K, Wakabayashi K, Takahashi H, et al. Cinnamoyl-CoA reductase, a key enzyme in lignin biosynthesis, is an effector of small GTPase Rac in defense signaling in rice. Proceedings of the National Academy of Sciences. 2006;103(1):230–235.

[13] Kopriva S, Koprivova A, Süss KH. Identification, cloning, and properties of cytosolic D-ribulose-5-phosphate 3-epimerase from higher plants. Journal of Biological Chemistry. 2000;275(2):1294–1299.

[14] Jelakovic S, Kopriva S, Süss KH, Schulz GE. Structure and catalytic mechanism of the cytosolic D-ribulose-5-phosphate 3-epimerase from rice. Journal of Molecular Biology. 2003;326(1):127–135.

[15] Nunes-Nesi A, Carrari F, Lytovchenko A, Smith AM, Loureiro ME, Ratcliffe RG, et al. Enhanced photosynthetic performance and growth as a consequence of decreasing mitochondrial malate dehydrogenase activity in transgenic tomato plants. Plant Physiology. 2005;137(2):611–622.

[16] Noguchi T, Hayashi S. Peroxisomal localization and properties of tryptophan aminotransferase in plant leaves. Journal of Biological Chemistry. 1980;255(6):2267–2269.

[17] Trovato M, Maras B, Linhares F, Costantino P. The plant oncogene rolD encodes a functional ornithine cyclodeaminase. Proceedings of the National Academy of Sciences. 2001;98(23):13449–13453.

[18] Pracharoenwattana I, Zhou W, Keech O, Francisco PB, Udomchalothorn T, Tschoep H, et al. Arabidopsis has a cytosolic fumarase required for the massive allocation of photosynthate into fumaric acid and for rapid plant growth on high nitrogen. The Plant Journal. 2010;62(5):785–795.

[19] Baum HJ, Madison JT, Thompson JF. Feedback inhibition of homoserine kinase from radish leaves. Phytochemistry. 1983;22(11):2409–2412.

[20] Amir R, Hacham Y, Galili G. Cystathionine γ-synthase and threonine synthase operate in concert to regulate carbon flow towards methionine in plants. Trends in Plant Science. 2002;7(4):153–156.

[21] Kürsteiner O, Dupuis I, Kuhlemeier C. The pyruvate decarboxylase1 gene of Arabidopsis is required during anoxia but not other environmental stresses. Plant Physiology. 2003;132(2):968–978.

[22] Zeh M, Casazza AP, Kreft O, Roessner U, Bieberich K, Willmitzer L, et al. Antisense inhibition of threonine synthase leads to high methionine content in transgenic potato plants. Plant Physiology. 2001;127(3):792–802.
